# Exposure to heat-stress environment affects the physiology, circulation levels of cytokines, and microbiome in dairy cows

**DOI:** 10.1038/s41598-018-32886-1

**Published:** 2018-10-02

**Authors:** Siyu Chen, Jian Wang, Dandan Peng, Gan Li, Jian Chen, Xianhong Gu

**Affiliations:** 1 State Key Laboratory of Animal Nutrition, Institute of Animal Science, Chinese Academy of Agricultural Sciences, Beijing, 100193 China; 2 Present Address: Institute of Animal Science, Chinese Academy of Agricultural Sciences, Yuanmingyuan West Road 2, Haidian strict, Beijing, 100193 China

## Abstract

The microbiome has emerged as a new player on behavior, physiology and stress because of its significant effects on the brain-gut axis. The aim of this study was to increase our understanding of brain-gut function in dairy cows. We investigated the effects of a heat-stress (HS) environment and individual differences of heat sensitivity (IH) on bovine physiological characteristics and microbial composition. Results indicate that both HS and IH increased rectal temperature (RT) (*P* < 0.05). An HS environment increased plasma, as well as milk cortisol and cytokines in plasma; however, it decreased plasma, and milk oxytocin, triiodothyronine, and thyroxine (*P* < 0.05) levels. Exposure to an HS environment reduced the diversity of the fecal microbial population, and resulted in a higher expression of diseases, the environmental adaptation pathway, and the immune related pathway, whereas it lowered the expression of metabolic pathways (*P* < 0.05). High heat sensitive cows have upregulated metabolisms, environmental adaptation and cellular process pathways, and a downregulated neurodegenerative disease pathway (*P* < 0.05). Thus, we conclude that exposure to an HS environment modulates physiological characteristics, which may interplay with microbial activity, and in turn, alter the circulation levels of cytokines, implicating the role of the brain-gut axis in dairy cows. The HS environment affected physiological characteristics, cytokine levels, and microbial composition, but IH influenced RT and fecal microbial functions.

## Introduction

The microbiome and its metabolites have a crucial role in the maintenance of host homeostasis^1^, and thus have become a booming area in both human^2^ and animal studies^3^. With respect to microbiome studies, the activity of the hypothalamus-pituitary-adrenal (HPA) axis of stress on microbial composition has been well reported^4^. Varying types of stress alter the microbial composition, such as social stress in mice^5^, heat stress (HS) in laying hens^6^, and weaning stress in dairy cows^7^. The gut microbiome is associated with the circulation levels of cytokines like interleukin-6^5^, and the modulation of the brain–gut axis function is subjected to stress and behavioral responses^8^. Notably, indiscriminate use of antibiotics in farm animals is associated with the development of antimicrobial resistance^9^. In addition, disturbances in the gut microbiome have been implicated in a wide range of disorders, including functional and inflammatory gastrointestinal disorders, obesity, and eating disorders in both animals and humans^10^. Therefore, the ability of the gut microbiome to communicate with the brain, and thus modulate physiology, behavior, and immune activity is emerging as an exciting concept. To date, there are many hypotheses implicating the brain-gut axis, which are generally influenced by bidirectional communication systems, such as the enteric nervous system; vagus, sympathetic, and spinal nerves; and humoral pathways, including cytokines, hormones, and neuropeptides as signaling molecules^11–13^. For example, having a normal gut microbiome moderates brain function and is essential for normal physiology and behaviors in mice^14,15^, and commensal microbes may play an important role in mastitis pathogenesis in dairy cows^16^. Contrarily, it is well known that HS has prolonged and negative effects on productivity^17^, fecundity^18^, and immunity^19^ in cows. Exposure to an HS environment has effects on physiological characteristics and circulation of the cytokines, as well as gut microbial composition, but there is still a lack of understanding of these interactions in dairy cows.

The temperature-humidity index (THI) is generally regarded as one of the indicators to evaluate HS^20^. However, since the THI is affected by many factors^21,22^, it is thus appropriate to evaluate HS at the flock level, rather than at the individual level. The panting score, which integrates the climatic variables like solar load and wind speed, as well as management factors (the effect of shade) and animal factors (genotype differences), is widely regarded as an accurate and reliable indicator of HS at the individual level^21^. Thus, by taking the characteristics of heat sensitivity into consideration, we investigated the effect of HS on the physiological characteristics and circulation levels of cytokines and microbial composition at a flock level (HS or none/slight-HS environments) and the individual level (high and low heat sensitivity). The aim was to better understand the effects of exposure to an HS environment on bovine physiology and immune activities, and how these responses interplay with the gut microbiome in dairy cows.

## Results

### Physiological characteristics

**Figure 1:**
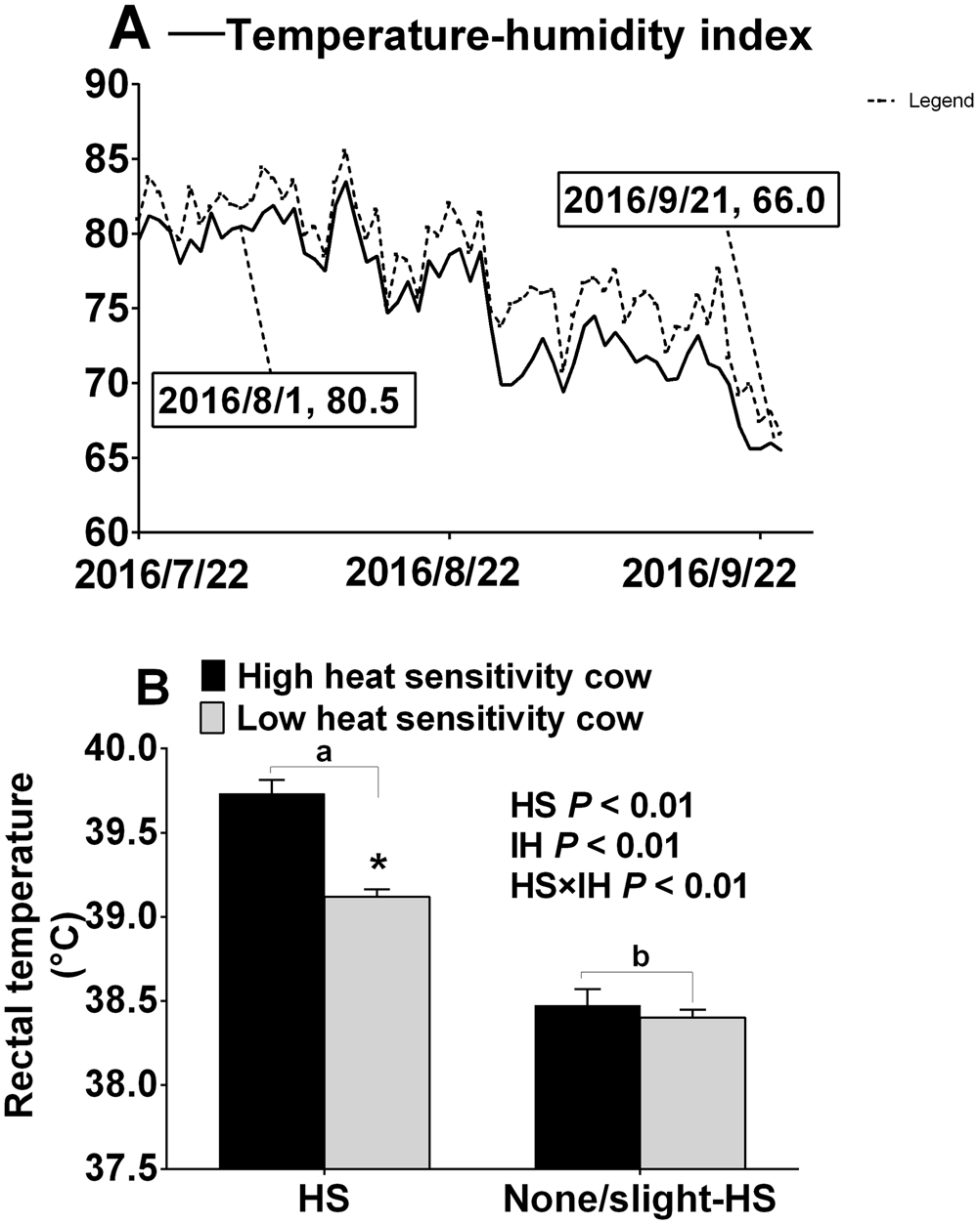
(**A**) Shows the temperature-humidity index (THI). (**B**) Shows rectal temperature of the four groups. Solid line represents the THI of 24 h data (144 values), and dotted line represents THI data based three hours (18 values) during 10:00 to 12:00 am. Data 2016/8/1 and 2016/9/21 represent the blood samplings days. HS means heat stress environment, IH means individual difference of heat sensitivity. *Means significant difference between High heat sensitivity cows under HS environment and low heat sensitivity cows under HS environment cows (*P* < 0.05). Values with different small letter (a,b) superscripts mean statistically different between HS and none/slight HS environment s (*P* < 0.05). n = 15 in each group.

The RT was affected by the HS environment and IH (both, *P* < 0.01) (Fig. 1B). High-heat-sensitivity cows under HS environment (H-HS) had higher RT than low-heat-sensitivity cows under HS environment (L-HS) (*P* < 0.01), and high-heat-sensitivity cows under none/slight-HS environment (H-nHS) and low-heat-sensitivity cows under non/slight-HS environment (L-nHS) did not show a difference. Further, RT of cows under HS environment (H-HS and L-HS) were higher than that in the cows under none/slight-HS environment (H-nHS and L-nHS) (*P* < 0.01), and the interaction between HS and IH was significant (*P* < 0.01).

**Figure 2:**
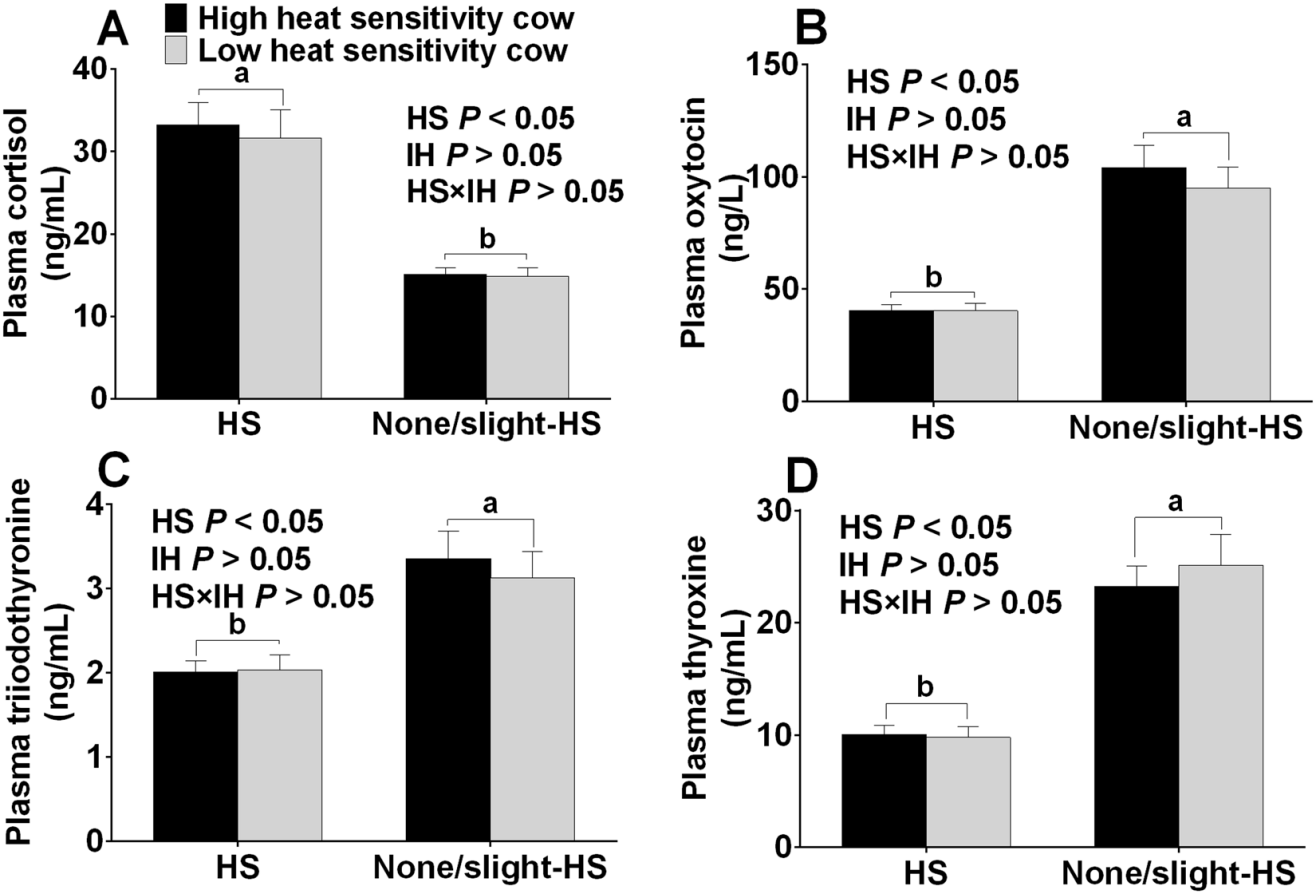
Plasma cortisol (**A**), oxytocin (**B**), triiodothyronine (**C**) and thyroxine (**D**) concentration of cows in the four groups. HS means heat stress environment, IH means individual difference of heat sensitivity. Values with different small letters (a,b) mean statistically different (*P* < 0.05). n = 15 in each group.

Plasma hormones were affected by the HS environment (*P* < 0.05) (Fig. 2). The plasma cortisol level of H-HS and L-HS cows were higher (*P* < 0.05), while oxytocin, triiodothyronine (T3) and thyroxine (T4) levels were lower than in the H-nHS and L-nHS cows (none/slight-HS environment) (*P* < 0.05) (Fig. 2). Neither the interaction between HS and IH, nor IH affected plasma cortisol, oxytocin, and T3 and T4 levels (*P* > 0.05).

**Figure 3:**
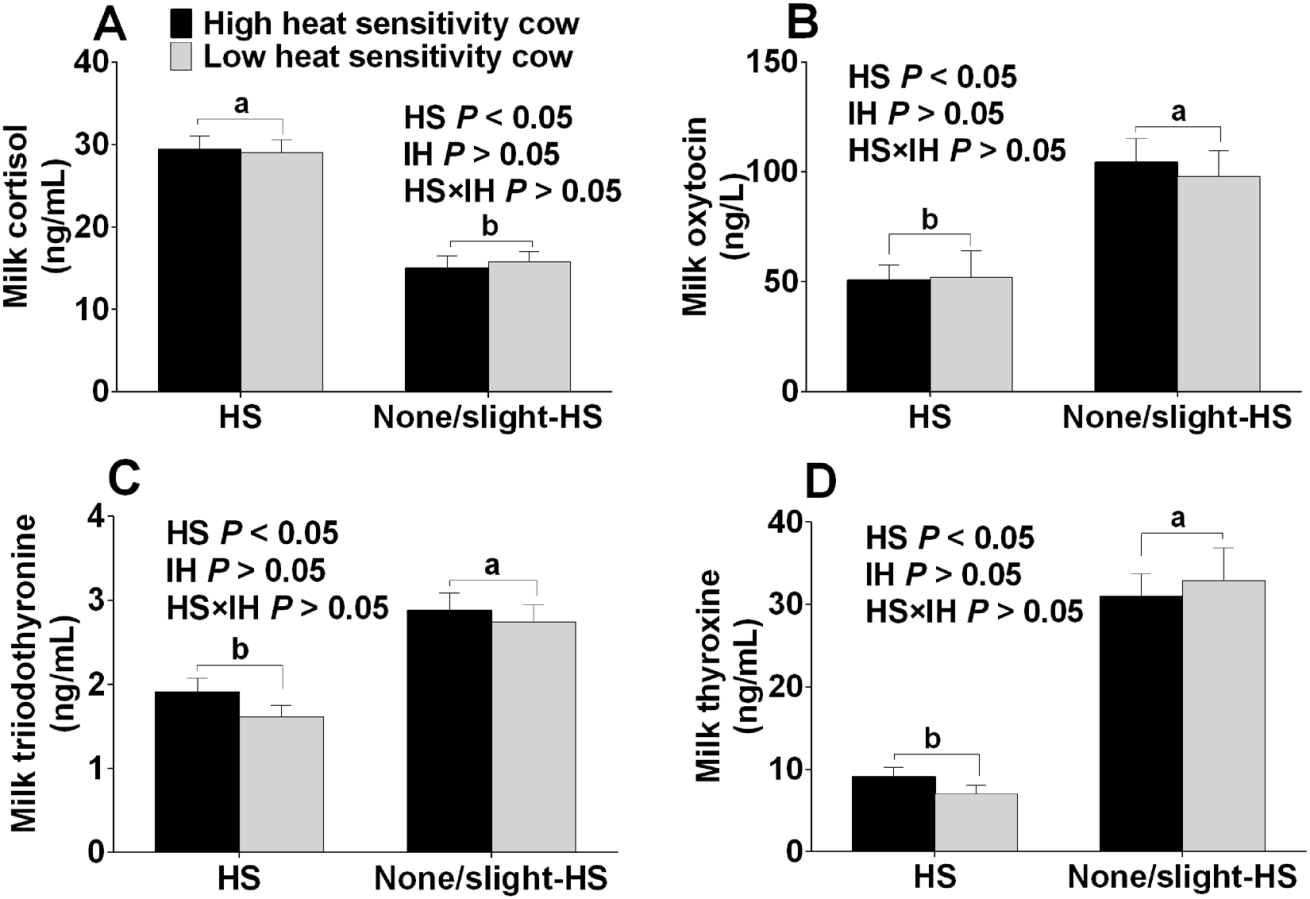
Milk cortisol (**A**), oxytocin (**B**), triiodothyronine (**C**) and thyroxine (**D**) concentration of cows in the four groups. HS means heat stress environment IH means individual difference of heat sensitivity. Values with different small letters (a,b) mean statistically different (*P* < 0.05). n = 15 in each group.

Milk hormones were also affected by an HS environment (*P* < 0.05) (Fig. 3). Milk cortisol of the H-HS and L-HS cows (HS environment) were higher (*P* < 0.05), while oxytocin, T3, and T4 levels were lower than in the H-nHS and L-nHS cows (none/slight-HS environment) (*P* < 0.05) (Fig. 3). Neither the interaction between HS and IH, nor IH affected milk cortisol, oxytocin, T3, and T4 levels (*P* > 0.05).

### Circulation levels of cytokines

**Figure 4:**
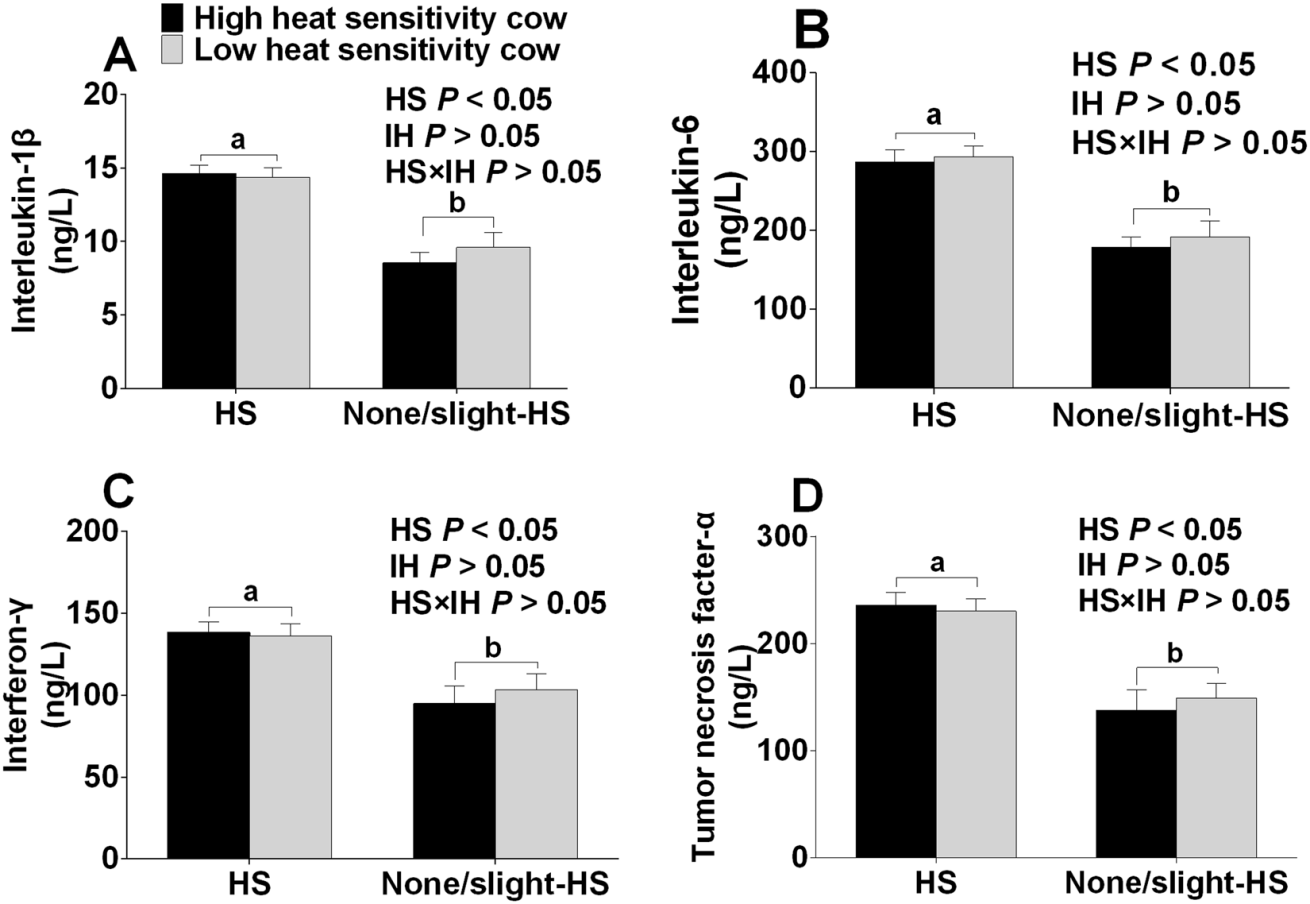
Plasma circulation levels of interleukin-1ß (**A**), interleukin–6 (**B**), interferon-γ (**C**), and tumor necrosis factor-α (**D**) of cows in four groups. HS means heat stress environment, IH means individual difference of heat sensitivity. Values with different small letters (a,b) mean statistically different (*P* < 0.05). n = 15 in each group.

Heat stress environment affected the circulation levels of cytokines in plasma (*P* < 0.05) (Fig. 4). The circulation levels of interleukin-1ß (IL-1ß), interleukin–6 (IL-6), interferon-γ (IFN-γ), and tumor necrosis factor-α (TNF-α) in plasma were all higher in the cows under an HS environment (H-HS and L-HS) than in the cows under a non/slight-HS environment (H-nHS and L-nHS) (All, *P* < 0.05). Neither interactions between HS and IH, nor IH affected these cytokine levels (Fig. 4).

### Feces microbial composition

**Figure 5:**
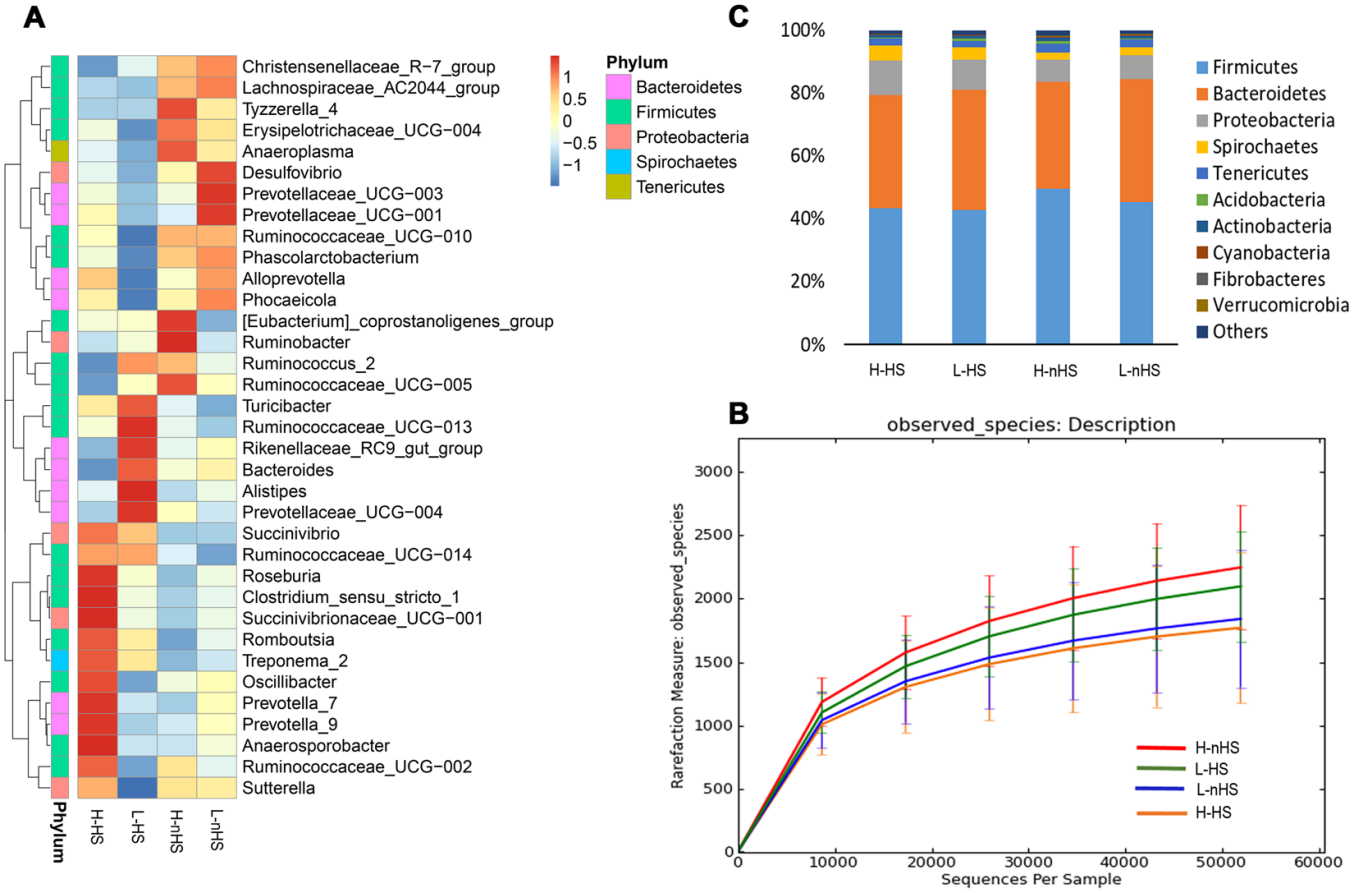
Feces microbial composition in the four groups. (**A**) Shows the heat map of the top 35 microbiome at genus level. (**B**) Shows the richness of feces microbial composition by observed species (richness of microbial composition: H-nHS > L-nHS, H-nHS > H-HS). (**C**) Shows the top 10 microbiome at phylum level. H-HS = high heat sensitivity cows under heat stress (HS) environment, L-HS = low heat sensitivity cows under HS environment (L-HS), H-nHS = high heat sensitivity cows under none/slight-HS environment, L-nHS = low heat sensitivity cows under non/slight-HS environment. n = 15 in each group.

Heat stress rather than IH affected the fecal microbial composition (Fig. 5A). The clusters of microbial composition of the H-HS and L-HS cows were similar, while the clusters of the H-nHS and L-nHS cows were similar. The microbial composition was richer in the H-nHS cows than in the L-nHS and H-HS cows (*P* < 0.05) and did not differ in other comparisons (Fig. 5B). The dominant microbes were *Firmicutes* and *Bacteroidetes* in all groups, of which the *Firmicutes* phylum seemed higher and *Bacteroidetes* phylum seemed lower in the H-nHS and L-nHS cows than in the H-HS and L-HS cows (Fig. 5C, *P* > 0.05).

**Figure 6:**
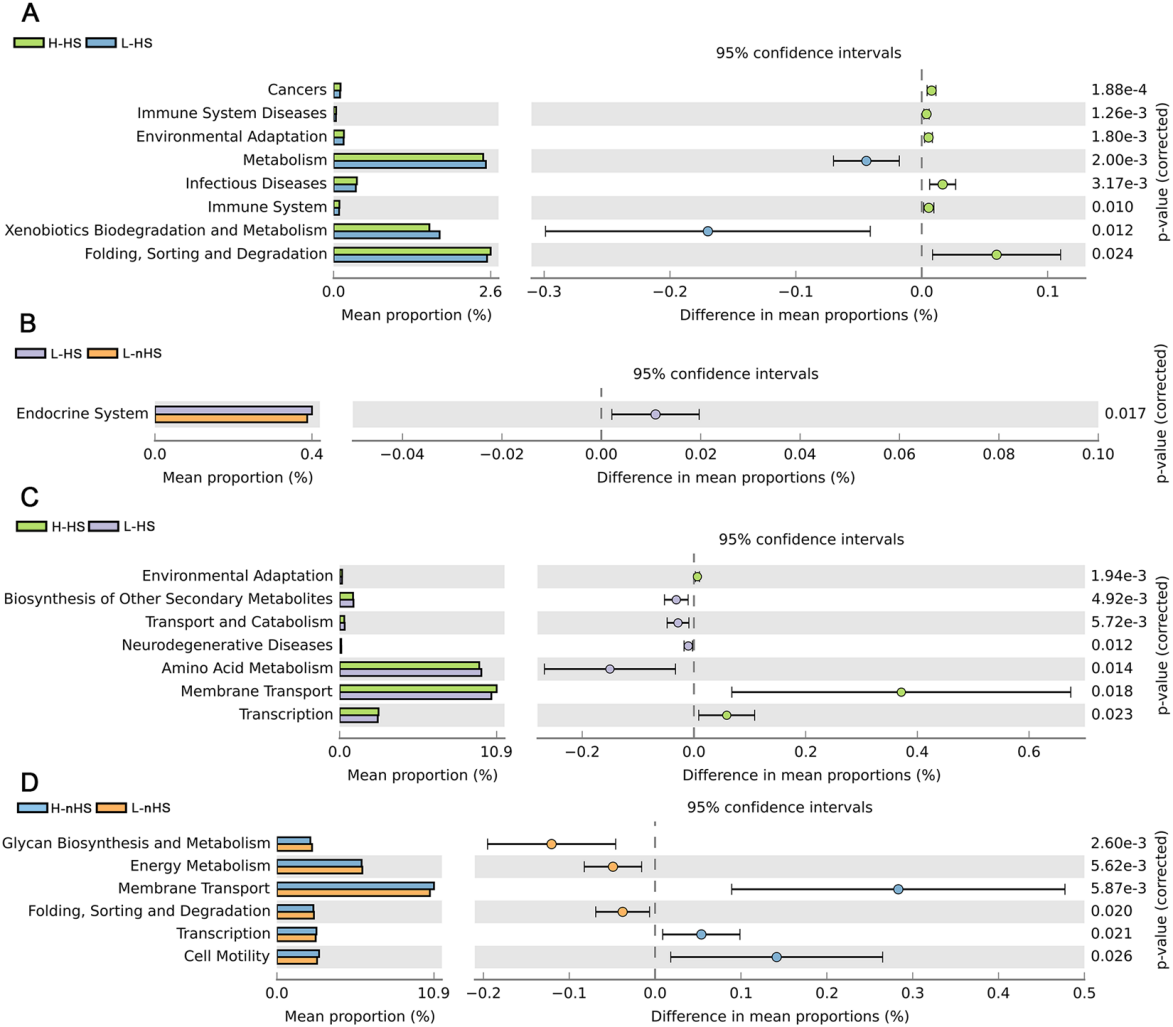
Feces microbiome functions between comparisons. (**A**) H-HS vs H-nHS; (**B**) L-HS vs L-nHS; (**C**) H-HS vs L-HS; (**D**) H-nHS vs L-nHS. H-HS = high heat sensitivity cows under heat stress (HS) environment, L-HS = low heat sensitivity cows under HS environment (L-HS), H-nHS = high heat sensitivity cows under none/slight-HS environment, L-nHS = low heat sensitivity cows under non/slight-HS environment. n = 15 in each group.

Heat stress environment resulted in different KEGG pathways of microbial function (*P* < 0.05) (Fig. 6A,B). The H-HS cows had upregulated expression of the pathways associated with diseases, including cancer, immune system disease, infectious disease, and genetic information processing (folding, sorting and degradation), and environmental adaptation pathways (*P* < 0.05, Fig. 6A). Whereas, they were downregulated in the metabolism pathways, sensory system pathway, and cell communication pathway (extremely low expression, and data are not shown) when compared with the H-nHS cows (*P* < 0.05, Fig. 6A). The L-HS cows had upregulated endocrine system pathway expression than the L-nHS cows (*P* < 0.05, Fig. 6B).

The IH affected microbial functions (*P* < 0.05) (Fig. 6C,D). When exposed to an HS environment, H-HS cows had an upregulated environmental adaptation, and cellular processes of membrane transport and transcription, while they had downregulated expression of the metabolism and neurodegenerative disease pathways than the H-nHS cows (*P* < 0.05, Fig. 6C). Under a none/slight-HS environment, the H-nHS cows had an upregulated expression of the cellular transport process pathway, while they had downregulated expression of the metabolic pathways (*P* < 0.05, Fig. 6D).

### Relationship of diversity of microbial composition to physiological characteristics and cytokines

**Figure 7:**
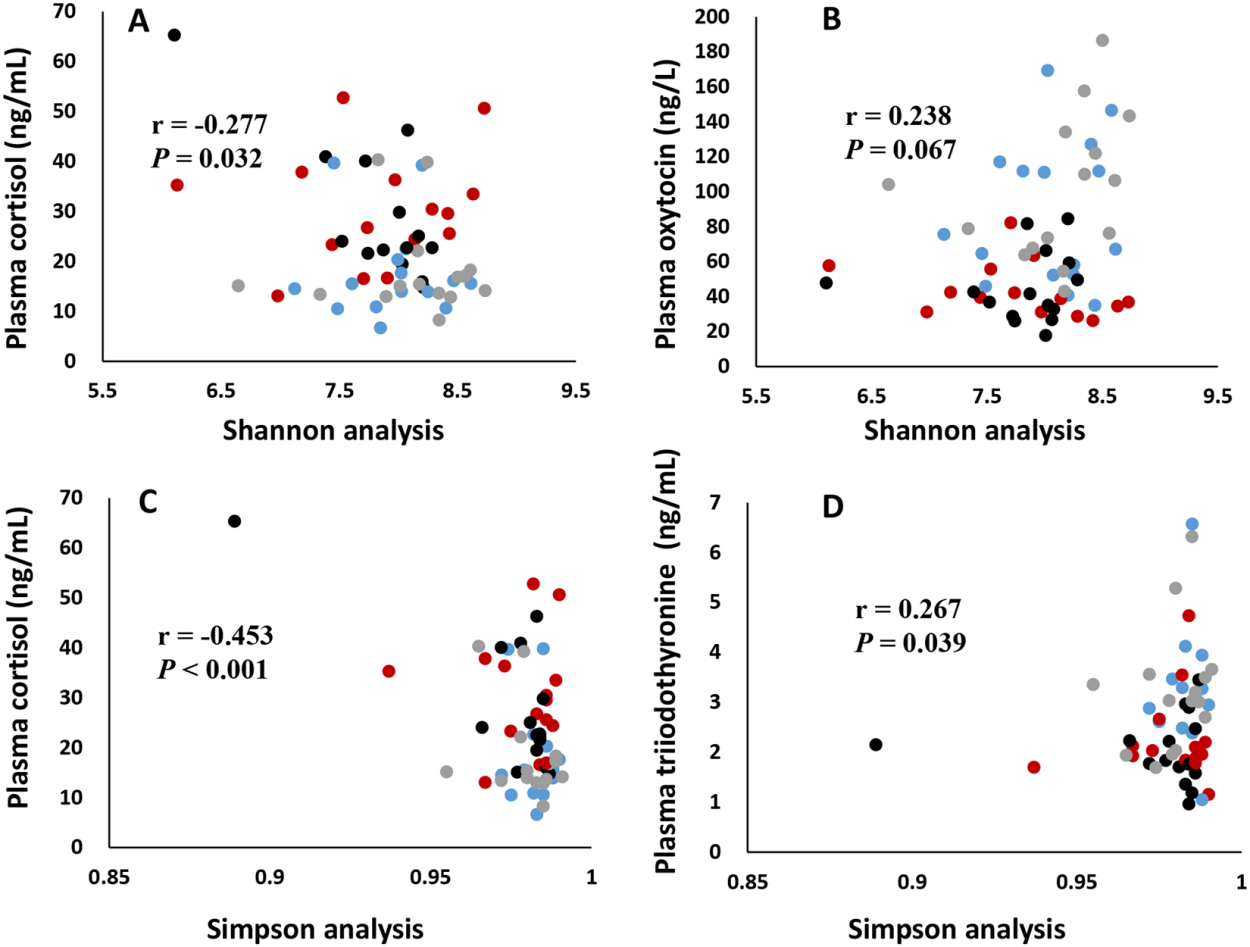
The relationship of feces microbial composition to plasma cortisol (**A**) and oxytocin (**B**) by Shannon analysis; and relationship of feces microbial composition to plasma cortisol (**C**) and triiodothyonine (**D**) by Simpson analysis. n = 60. Red circles represent high heat sensitivity cows under heat stress (HS) environment (H-HS), black circles represent low heat sensitivity cows under HS environment (L-HS), gray circles represent high heat sensitivity cows under none/slight-HS environment (H-nHS), and blue circles represent low heat sensitivity cows under non/slight-HS environment (L-nHS).

**Figure 8:**
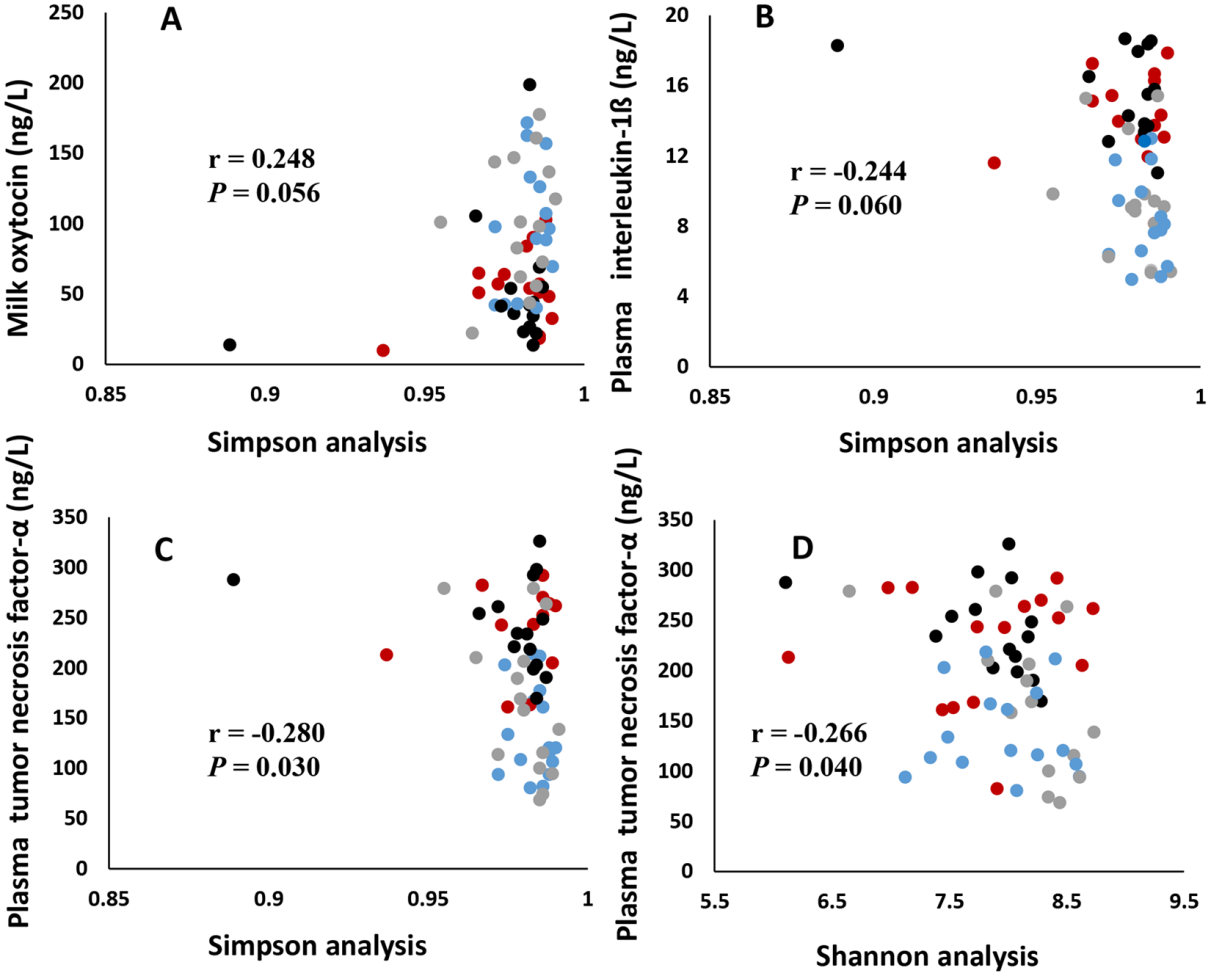
The relationship of feces microbial composition to milk oxytocin (**A**) and plasma interleukin-1β (**B**) by Simpson analysis; and relationship of feces microbial composition to plasma tumor necrosis factor-α by Simpson (**C**) and Shannon (**D**) analysis. n = 60. Red circles represent high heat sensitivity cows under heat stress (HS) environment (H-HS), black circles represent low heat sensitivity cows under HS environment (L-HS), gray circles represent high heat sensitivity cows under none/slight-HS environment (H-nHS), and blue circles represent low heat sensitivity cows under non/slight-HS environment (L-nHS).

The diversity of microbial composition was negatively correlated with plasma cortisol, as assessed using both the Shannon (r = −0.277, *P* = 0.032; Fig. 7A) and Simpson indices (r = −0.453, *P* < 0.001; Fig. 7C). Furthermore, microbial composition had a trend of positive correlation with plasma oxytocin, assessed using Shannon diversity index (r = 0.238, *P* = 0.067; Fig. 7B) but not using Simpson’s diversity index, and it was also positively correlated with plasma T3, assessed using Simpson’s index (r = 0.267, *P* = 0.039; Fig. 7D) but not using Shannon index. Additionally, using Shannon’s index, microbial composition was found to be positively correlated with milk oxytocin levels (r = 0.248, *P* = 0.056; Fig. 8A). Using Simpson’s index, a negative correlation was found between the microbial composition and circulation levels of IL-1ß (r = −0.244, *P* = 0.060; Fig. 8B); a negative trend of microbial composition with TNF-α was found using both Simpson’s index (r = −0.280, *P* = 0.030; Fig. 8C) and Shannon’s index (r = −0.266, *P* = 0.040; Fig. 8D). Otherwise, no significant correlations were found between microbial composition and other plasma and milk physiological characteristics, as well as other cytokines.

## Discussion

The microbiome is a new indicator of the various effects on the brain-gut axis. Although the mechanism of this axis has not yet been completely understood, it is hypothesized that stressor-induced alterations of the gut microbiome result in the translocation of bacterial products to act as stimuli for the innate immune system^5^. Our study emphasizes the effect of the HPA axis on bovine physiological characteristics, immune activity, and microbiome in response to HS environments.

As a widely used indicator to evaluate HS^23,24^, the RT is higher under HS environment than under none/slight HS environment, suggesting that H-HS and H-nHS cows suffer from HS. Heat stress results in higher cortisol concentrations in both the plasma and milk. Cortisol is one of the stress indicators modulated by the HPA axis in dairy cows and horses^25^, and our results are consistent with previous studies^26,27^. T3 and T4, as modulators, play a major role in the growth and development of the brain and central nervous system^28^, and are known to decrease under HS simultaneously with a decrease in feed intake^29,30^. They are both lower in cows under an HS environment than under a none/slight environment, which is similar to the lower values in summer (higher ambient temperature) vs those in winter (lower ambient temperature)^31^. Additionally, oxytocin decreased when dairy cows were exposed to psychological stressors^32^. The higher oxytocin values in cows under a none/slight HS environment than under an HS environment indicated that suffering resulted from HS. All these findings suggest that the exposure to an HS environment alters physiological characteristics in cows.

The circulation levels of IL-1ß, IL-6, IFN-γ, and TNF-α, are all higher in the HS-exposed cows (H-HS and L-HS cows) than in the cows exposed to none/slight-HS environments (H-nHS and L-nHS cows), which suggests the possibility of an inflammatory condition resulting from the HS environment^33^. This result was supported by the fact that oxidative stress caused inflammation^34^, and plasma TNF-α and IL-6 were shown to increase under long-term heat stress in dairy cows^35^.

The microbial population is richer in cows exposed to none/slight-HS environments (H-nHS) than those exposed to an HS condition (H-HS), which is similar with results of previous studies indicating that heat stress changed the rumen microbial population of dairy cows^36^ and reduced alpha diversity of the gut microbiome in mice^5,37^. The latter study further revealed that circulating levels of IL-6 and CCL2 correlated with stressor-induced changes in *Coprococcus* spp., *Pseudobutyrivibrio* spp., and *Dorea* spp. In our study, microbial composition was positively correlated with oxytocin and T3, and negatively correlated with the stress indicator, cortisol, as well as with the inflammatory mediators, IL-1ß and TNF-α. These correlations suggest that HS induces poorer microbial composition and provides further evidence for the possible role of the brain-gut axis in the physiological characteristics and immune activities in dairy cows.

The dominant microbes are *Firmicutes*, *Bacteroidetes*, and *Proteobacteri*a, which were similar with a previous study, regardless of the age of dairy cows^38^. The gut microbiome is crucial in optimizing dairy cow health and production efficiency and facilitated the development of gut tissue and immune system^39^. The dynamic balances of ruminal and gut microbiomes, host physiology, and diet influence the initial acquisition and eventual stability of the rumen and gut ecosystems^40^. The above studies emphasize that exposure to an HS environment results in the upregulation of diseases, but downregulation of metabolic pathways (H-HS vs H-nHS). Thus, these findings suggest that an HS environment affects microbial activities linking to potential diseases and lower metabolisms in cows. The upregulated immune system (H-HS vs H-nHS) and endocrine system pathways (L-HS vs L-nHS) under the HS rather than the none/slight-HS condition, provides evidence that exposure to HS leads to the alteration of microbial activities, which may in turn enhances plasma circulation levels of cytokines in cows. This may be supported by previous findings that the microbiome affected adaptive immune regulation linking to inflammatory diseases, such as asthma, in animals^41^ and humans^42^. When exposed to HS, the heat shock transcription factor 1 is transcribed in the cell and plays an important role in the onset of elevated cell temperature^43^. This study explains the upregulated transcription pathway found in H-HS cows than in the H-nHS cows, although we did not obtain gene expression of heat shock transcription factor 1 in this study. The upregulated environmental adaptation pathway in H-HS as opposed to that in H-nHS cows, profiles the response of cows coping with the HS condition. It is possible that high-heat-sensitivity cows readily tolerated high temperatures, for which cows would modulate physiological and biochemical adaptations to prevent overheating when temperatures were above the thermoneutral zone^44^.

The high-heat-sensitivity cows have a higher RT than low-heat-sensitivity cows under both HS and none/slight-HS environments (H-HS > L-HS; H-nHS > L-nHS), besides increased respiratory rate^45^, and decrease of T3 and T4^31^ concentrations to maintain homeostasis and prevent overheating. The increased respiratory rate relates to energy consumption when animals cope with the ambient environment^46,47^. This explains the downregulated pathways of metabolisms, especially energy and amid acid metabolisms in H-HS and H-nHS cows as opposed to L-HS and L-nHS cows. The upregulated pathways of environmental adaptation and membrane transport in high- rather than in low-heat-sensitivity cows under the HS environment (H-HS vs L-HS) may be modulated by the same mechanism as mentioned above, wherein HS cows readily tolerated high temperature by modulating physiological and biochemical adaptations to the thermal environment. This is also seen as evidence from the upregulated cell motility and transcription pathways in H-nHS cows rather than in L-nHS cows, in which a number of anomalies in cellular function are induced^48^ alongside the changes in gene transcription and metabolism shifts when coping with the thermal environment in dairy cows^49,50^. However, it is not clear why the neurodegenerative disease pathway is upregulated in L-HS cows rather than in H-HS cows. In addition, the physiological characteristics and circulation levels of cytokines seem to be affected by HS rather than IH (neither differs between H-HS and L-HS, nor between H-nHS and L-nHS). These findings are further confirmed by the similarities of the microbial composition cluster between high- and low-heat-sensitivity cows under both the HS and none/slight-HS environments.

It has long been hypothesized that stressor-induced alterations of the gut microbiome results in the altered physiology and immunity. Our study emphasizes the effect of HS on physiological characteristics and circulation levels of immune activity and the microbiome. We conclude that the exposure to HS modulates physiology and immunity, which may interplay with the microbial activities and alter circulation levels of cytokines, asserting the role of the brain-gut axis in dairy cows. HS, rather than IH, affects physiological characteristics, cytokines, and microbial composition, but IH influences RT and fecal microbial functions, implying there is a difference at the individual level within the flock.

## Methods

### Animals and groups

All methods and animal care were performed in accordance with the relevant guidelines and regulations of the Institute of Animal Science, Chinese Academy of Agricultural Sciences. This study was carried out at a private farm in Shunyi district, Beijing from July to September in 2016; however, the rations were not changed during June to October, covering the experimental period. Chinese Holstein dairy cows in the same barn were used. The barn (260 m × 15 m) having the capacity of 280 cows, was concrete-floored without an outdoor area, where every cow had more than one bed with litter of rice husk. Cows were fed total mixed ration three times daily, at 07:00 am, 14:00 pm, and 19:00 pm, with free accesses to water and diet, and were milked three times daily, at 08:00 am, 15:10 pm, and 21:40 pm. Feces were removed by an automatic shaving machine.

The test initially took place over five consecutive days to select the experimental cows under high THI conditions (THI = 80 ± 1.15 (SD)). The temperature (T) and relative humidity (RH) were recorded every 10 min by automatic temperature and humidity recorder (RC-4, China) during testing days. THI was calculated based on 144 averaged values (24 h) of T and RH by the formula: THI = (1.8 × T + 32) − [(0.55 − 0.0055 × RH) × (1.8 × T−26.8)] (NRC, 1971). During these five days, all 280 cows were scored by the panting score, measured 15 times a day, to classify the character of heat sensitivity. For panting score, a four-grade evaluation system was used^21^ for dairy cows, where 0 presented low heat sensitivity: Score 0 = Normal breath with slight panting; 1 = Slow breath, along with the movements of abdomen and hindquarters, but not spine and head; 2 = Relatively quick breath, along with movements of hindquarters, spine and head, and occasional snot; 3 = Quick breath, with mouth opening and tongue extending, and additional snot and drool. Taking the milk yield, parity, and days in milk into consideration, and based on the panting score high (n = 15) and low (n = 15), heat sensitive cows were selected as experimental cows (see Table 1).

**Table 1:** The basic information of experimental cows.

Items	High heat sensitivity cows (n = 15)	Low heat sensitivity cows (n = 15)	SEM	*P*-Value
Panting score^a^	1.68	1.14	0.103	<0.01
Milk yield (kg/day)	33.8	37.1	1.528	0.15
Parity	3.53	3.13	0.350	0.43
Days in milk	106.5	92.3	9.337	0.29

^a^Average value of the 15 times data during the consecutive five days.

Accordingly, previous studies suggested cows suffer HS when the thermal temperature is above the thermoneutral zone, such as THI higher than 72^51^, higher than 68, 69, 72^52^ and higher than 68^53^. Therefore, measurement was taken under high THI (80.5) suggesting a HS environment and low THI (66.0) indicating a none/slight-HS environment. Figure 1A shows averaged 24 h and 3 h (10:00 am to 12:00 am) THI during the experimental period. Thus, four experimental groups were merged: the high-heat-sensitivity cows under heat stress environment (H-HS, n = 15); low-heat-sensitivity cows under heat stress environment (L-HS, n = 15); high-heat-sensitivity cows under none/slight heat stress environment (H-nHS, n = 15); and low-heat-sensitivity cows under none/slight heat stress environment (L-nHS, n = 15).

### Samplings

#### Physiological characteristics and circulation levels of cytokines

Blood samples (10 mL in each) were collected at 11:00 am (neither the feeding nor milking time) under HS (THI = 80.5 ± 1.8 (SD)) and none/slight-HS (THI = 66.0 ± 3.4 (SD)) conditions, from the coccygeal vein using vacutainer tubes (BD Biosciences, San Jose, CA). Plasma was separated through centrifugation at 3,000 × g for 10 min at 4 °C and stored at −20 °C for subsequent detection. Cortisol (ng/mL), oxytocin (ng/L), triiodothyronine T3 (ng/mL), T4 (ng/mL), and circulation levels (ng/L) of IL-1ß, IL-6, IFN-γ, and TNF-α were measured using the commercial assay ELISA kits (XL-Eb0187) from Shanghai Xinle Biotechnology, Ltd. (Shanghai, China).

Milk samples (10 mL in each) were collected during milking at 3:10 pm on the day before blood samplings days under HS (THI = 80.3 ± 3.4 (SD)) and less/non-HS (THI = 65.5 ± 5.3 (SD)) environments, respectively. The skimmed milk was obtained through two consecutive centrifugations at 4,000 × g for 20 min at 4 °C and stored at −20 °C for the measurement of cortisol, oxytocin, T3 and T4 levels by ELISA kits (XL-Eb0142) from Shanghai Xinle Biotechnology, Ltd. (Shanghai, China).

RT (°C) was manually measured by a rectal thermometer (Wuhan, China) at 10 cm insertion to minimize bias^54^ at 23:00 pm on the day after blood sampling days, under HS (THI = 80.2 ± 2.6 (SD)) and none/slight-HS (THI = 65.5 ± 2.7 (SD)) environments.

#### Feces microbiome

On the day after blood sampling days, 20 g rectal feces were collected immediately after measuring RT and stored at −20 °C for DNA extraction. Total genome DNA was extracted using QIAamp Fast DNA Stool Mini Kit (QIAGEN, Hilden, Germany). The V4 region of 16 S rDNA was amplified by using the 515 f/806r primer set. All PCR reactions were conducted using Phusion® High-Fidelity PCR Master Mix (NEB, Beverly, MA, USA). PCR products were purified using the QIAquick Gel Extraction Kit (QIAGEN, Hilden, Germany). The libraries were generated using TruSeq® DNA PCR-Free Sample Preparation Kit (Illumina, San Diego, CA, USA) and sequencing was conducted on Illumina HiSeq. 2500 platform.

Paired-end reads were merged using FLASH v1.2.7^55^. Chimeric sequences were removed using the UCHIME algorithm^56^. Quality filtering on the raw tags was performed by QIIME v1.7.0^57^. Operational Taxonomic Unit (OTUs) was assigned using Uparse v7.0.1001^58^ with a 97% similarity threshold. Taxonomic annotation was performed by comparing sequences to the GreenGenes Database. The functions of microbial composition were predicted using online PICRUSTs (http://huttenhower.sph.harvard.edu/galaxy). The results were analyzed by the two-tailed Welch’s t-test and the KEGG pathways, with Store FDR-corrected *P*-value (*q*-value) < 0.05 were regarded as significant, and then were depicted using the STAMP software.

### Statistical analyses

All data were analyzed as mean ± SE by SAS 9.3 (SAS Institute Inc., Cary N.C., USA) unless otherwise noted. To analyze the effects on physiological characteristics and cytokines, the following model of PROC GLM was used:

$${\rm{Yijk}}={\rm{\mu }}+{\rm{Ai}}+{\rm{Bj}}+({\rm{AB}})\,{\rm{ij}}+{{\rm{b}}}_{1}{\rm{Xij}}+{{\rm{b}}}_{2}{\rm{Xij}}+{\rm{\varepsilon }}\mathrm{ijk},{\rm{i}}=1,2;\,{\rm{j}}=1,2;\,{\rm{k}}=1,\ldots ,15$$

where Yijk is the observation k at the level i HS conditions and the level j heat sensitivity, μ is the overall mean, Ai is the effect of level i HS conditions, Bj is the effect of level j heat sensitivity, (AB)ij is the effect of the interaction of level i HS conditions with level j heat sensitivity, b_1_Xij is the covariant factor of DIM, b_2_Xij is the covariant factor of milk yield, εijk is the random error with mean 0 and variance σ2, and k is the cow. When significant interactions (*P* < 0.05) were detected, adjustments to the level of significance were used to account for the comparisons between HS conditions as well as between high and low sensitive cows using Tukey-Kramer adjustments. In relation to the gut microbiome, alpha diversity analyses of Shannon, and Simpson indices were analyzed by the ANOVA test. Pearson’s correlation coefficients (r) were calculated for relationships between physiological characteristics, cytokines, and richness of microbial composition. Values with *P* < 0.05 were regarded as statistically significant.

### Ethics statement

The experimental protocols were approved (Approval number: 2016IAS018) by the Experimental Animal Care and Committee of Institute of Animal Science, Chinese Academy of Agricultural Sciences.

## Data Availability

The datasets generated and/or analyzed during the current study are available from the corresponding author on reasonable request.

## References

[1] Clemente JC, Ursell LK, Parfrey LW, Knight R (2012). The impact of the gut microbiota on human health: an integrative view. Cell.

[2] Planer JD (2016). Development of the gut microbiota and mucosal IgA responses in twins and gnotobiotic mice. Nature.

[3] Lee WJ, Hase K (2014). Gut microbiota-generated metabolites in animal health and disease. Nat. Chem. Biol..

[4] Tannock GW, Savage DC (1974). Influences of dietary and environmental stress on microbial populations in the murine gastrointestinal tract. Infect. Immun..

[5] Bailey MT (2011). Exposure to a social stressor alters the structure of the intestinal microbiota: implications for stressor-induced immunomodulation. Brain Behav. Immun..

[6] Zhang P (2016). Probiotic mixture ameliorates heat stress of laying hens by enhancing intestinal barrier function and improving gut microbiota. Ital. J. Anim. Sci..

[7] Davis MY (2016). Rapid change of fecal microbiome and disappearance ofClostridium difficilein a colonized infant after transition from breast milk to cow milk. Microbiome.

[8] Rhee SH, Pothoulakis C, Mayer EA (2009). Principles and clinical implications of the brain-gut-enteric microbiota axis. Nat. Rev. Gastroenterol. Hepatol..

[9] Antonopoulos DA (2008). Decreased diversity of the fecal microbiome in recurrent clostridium difficile: associated diarrhea. J. Infect. Dis..

[10] Mayer EA (2011). Gut feelings: the emerging biology of gut-brain communication. Nat. Rev. Neurosci..

[11] Cryan JF, O’Mahony SM (2011). The microbiome-gut-brain axis: from bowel to behavior. Neurogastroenterol. Motil..

[12] Chen X, D’Souza R, Hong ST (2013). The role of gut microbiota in the gut-brain axis: current challenges and perspectives. Protein Cell.

[13] Mayer EA, Knight R, Mazmanian SK, Cryan JF, Tillisch K (2014). Gut microbes and the brain: paradigm shift in neuroscience. J. Neurosci..

[14] Heijtz RD (2011). Normal gut microbiota modulates brain development and behavior. Proc. Natl. Acad. Sci. USA.

[15] Cryan JF, Dinan TG (2012). Mind-altering microorganisms: the impact of the gut microbiota on brain and behaviour. Nat. Rev. Neurosci..

[16] Ecr B (2017). Milk microbiome and bacterial load following dry cow therapy without antibiotics in dairy cows with healthy mammary gland. Sci. Rep..

[17] Ray DE, Halbach TJ, Armstrong DV (1992). Season and lactation number effects on milk production and reproduction of dairy cattle in Arizona. J. Dairy Sci..

[18] Rensis FD (2002). Fertility in postpartum dairy cows in winter or summer following estrus synchronization and fixed time AI after the induction of an LH surge with GnRH or hCG. Theriogenology.

[19] Koch, F., Albrecht, E. & Kuhla, B. The gut barrier integrity and mucosal immune response after heat stress in the jejunum of dairy cows. Abstract retrieved from conference of Gfe Göttingen (2017).

[20] Preez JHD, Hattingh PJ, Giesecke WH, Eisenberg BE (1990). Heat stress in dairy cattle and other livestock under Southern African conditions. III. Monthly temperature-humidity index mean values and their significance in the performance of dairy cattle. Onderstepoort J. Vet. Res..

[21] Gaughan JB, Mader TL, Holt SM, Lisle A (2008). A new heat load index for feedlot cattle. J. Anim. Sci..

[22] Dikmen S, Hansen PJ (2009). Is the temperature-humidity index the best indicator of heat stress in lactating dairy cows in a subtropical environment?. J. Dairy Sci..

[23] Younes RB (2011). Hormonal (Thyroxin, Cortisol) and Immunological (Leucocytes) Responses to Cistern Size and Heat Stress in Tunisia. J. Life Sci..

[24] Ammer S, Lambertz C, Gauly M (2016). Comparison of different measuring methods for body temperature in lactating cows under different climatic conditions. J. Dairy Res..

[25] Schwinn AC, Sauer F, Gerber V, Bruckmaier RM, Gross JJ (2018). Technical note: Free and bound cortisol in plasma and saliva during ACTH challenge in dairy cows and horses. J. Anim. Sci..

[26] Abilay TA, Mitra R, Johnson HD (1975). Plasma cortisol and total progestin levels in Holstein steers during acute exposure to high environmental temperature (42 C) conditions. J. Anim. Sci..

[27] Verkerk GA, Phipps AM, Matthews LR (1996). Milk cortisol concentrations as an indicator of stress in lactating dairy cows. Proc. N. Z. Soc. Anim. Prod..

[28] Cann SAH (2006). Hypothesis: dietary iodine intake in the etiology of cardiovascular disease. J. Am. Coll. Nutr..

[29] O’Brien MD, Rhoads RP, Sanders SR, Duff GC, Baumgard LH (2010). Metabolic adaptations to heat stress in growing cattle. Domest. Anim. Endocrin..

[30] Hansen PJ (2009). Effect of heat stress on mammalian reproduction. Phil. Trans. R. Soc. Lond. B. Biol. Sci..

[31] Lopez E (2017). Stress-related hormonal alterations, growth and pelleted starter intake in pre-weaning Holstein calves in response to thermal stress. Int. J. Biometeorol..

[32] Sutherland MA, Tops M (2014). Possible involvement of oxytocin in modulating the stress response in lactating dairy cows[J]. Front. Psychol..

[33] Brook I (2008). Microbiology and management of abdominal infections. Dig. Dis. Sci..

[34] Ibrahim HMM (2015). Cytokine response and oxidative stress status in dairy cows with acute clinical mastitis. J. Dairy Vet. Anim. Res..

[35] Min L (2016). Long-term heat stress induces the inflammatory response in dairy cows revealed by plasma proteome analysis. Biochem. Biophys. Res. Commun..

[36] Yadav B (2013). Impact of heat stress on rumen functions. Vet. World.

[37] Bailey MT (2010). Stressor exposure disrupts commensal microbial populations in the intestines and leads to increased colonization by Citrobacter rodentium. Infect. Immun..

[38] Jami E, Israel A, Kotser A, Mizrahi I (2013). Exploring the bovine rumen bacterial community from birth to adulthood. Isme J..

[39] Macpherson AJ, Harris NL (2004). Interactions between commensal intestinal bacteria and the immune system. Nat. Rev. Immunol..

[40] Jami E, Mizrahi I (2012). Composition and similarity of bovine rumen microbiota across individual animals. PLoS ONE.

[41] Russell SL (2013). Early life antibiotic-driven changes in microbiota enhance susceptibility to allergic asthma. Embo Rep..

[42] Huffnagle GB (2010). The microbiota and allergies/asthma. PLoS Pathog..

[43] Trinklein ND, Murray JI, Hartman SJ, Botsein. D, Myers RM (2004). The role of heat shock transcription factor 1 in the genome-wide regulation of the mammalian heat shock response. Mol. Biol. Cell.

[44] Gebremedhin KG, Wu B (2002). Simulation of sensible and latent heat losses from wet-skin surface and fur layer. J. Therm. Biol..

[45] da Costa ANL, Feitosa JV, Montezuma PA, de Souza PT, de Araújo AA (2015). Rectal temperatures, respiratory rates, production, and reproduction performances of crossbred Girolando cows under heat stress in northeastern Brazil. Int. J. Biometeorol..

[46] West JW (2003). Effects of heat-stress on production in dairy cattle. J. Dairy Sci..

[47] Wolp RC (2012). Soybean oil and crude protein levels for growing pigs kept under heat stress conditions. Livest. Sci..

[48] Sonna LA, Fujita J, Gaffin SL, Lilly CM (2002). Effects of heat and cold stress on mammalian gene expression. J. Appl. Physiol..

[49] Lanks KW (1986). Modulators of the eukaryotic heat shock response. Exp. Cell Res..

[50] Lindquist S (1986). The heat-shock response. Annu. Rev. Biochem..

[51] Ravagnolo O, Miztal I (2000). Genetic component of heat stress in dairy cattle, parameter estimation. J. Dairy Sci..

[52] Bryant JR, López‐Villalobos N, Pryce JE, Holmes CW, Johnson DL (2007). Quantifying the effect of thermal environment on production traits in three breeds of dairy cattle in New Zealand. New Zeal. J. Agr. Res..

[53] Zimbelman, R. B. *et al*. A re-evaluation of the impact of temperature humidity index (THI) and black globe humidity index (BGHI) on milk production in high producing dairy cows. *Proc. Southwest Nutr. Man. Conf*. 158-168 (2009).

[54] Burfeind O, von Keyserlingk MA, Weary DM, Veira DM, Heuwieser W (2010). Short communication: repeatability of measures of rectal temperature in dairy cows. J. Dairy Sci..

[55] Magoc T, Salzberg SL (2011). FLASH: fast length adjustment of short reads to improve genome assemblies. Bioinformatics.

[56] Caporaso JG (2010). QIIME allows analysis of high-throughput community sequencing data. Nat. Methods.

[57] Edgar RC, Haas BJ, Clemente JC, Quince C, Knight R (2011). UCHIME improves sensitivity and speed of chimera detection. Bioinformatics.

[58] Edgar RC (2013). UPARSE: highly accurate OTU sequences from microbial amplicon reads. Nat. Methods.

